# Haplotype-tagging interleukin-10 promoter polymorphism is associated with reduced risk of retinal artery occlusion

**Published:** 2007-04-04

**Authors:** Martin Weger, Iris Steinbrugger, Yosuf El-Shabrawi, Beate Julia Wegscheider, Wolfgang Weger, Wilfried Renner, Otto Schmut, Anton Haas

**Affiliations:** 1 Department of Ophthalmology, Medical University of Graz, Austria; 2 Department of Dermatology, Medical University of Graz, Austria; 3 Clinical Institute of Medical and Chemical Laboratory Diagnostics, Medical University of Graz, Austria

## Abstract

**Purpose:**

Pro- and anti-inflammatory cytokines, including interleukin 10 (IL-10), play an essential role in atherogenesis. Increased IL-10 production has been found among carriers of the IL10 [TCATA] haplotype, which is formed by five polymorphisms at position -3575, -2763, -1082, -819, and -592 in the promoter region of the IL10 gene. Due to linkage disequilibrium, the presence of the [TCATA] haplotype can be unequivocally determined by analysis of the IL10-592C>A polymorphism. The purpose of the present study was to investigate a hypothesized association between the haplotype-tagging IL10 -592C>A polymorphism and the presence of retinal artery occlusion (RAO).

**Methods:**

The present case-control study was comprised of 194 patients with RAO and 257 normal control subjects. Genotypes of the IL10 -592C>A polymorphism were determined by fluorogenic exonuclease (TaqMan) assay.

**Results:**

Carriers of the IL10 -592A-allele, indicating the presence of the IL10 [TCATA] haplotype, were found significantly more often in controls than among patients with RAO (48.6% versus 36.1%; p=0.008). In a logistic regression analysis after adjusting for age, sex, arterial hypertension, diabetes mellitus, hypercholesterolemia, and smoking habits, carriage of the IL10 -592A-allele was associated with an odds ratio of 0.65 (95% CI: 0.44-0.97) for RAO.

**Conclusions:**

Our data suggest that the IL10 [TCATA] haplotype, identified by the presence of the IL10 -592A-allele, may exert a protective effect against RAO.

## Introduction

Retinal artery occlusion (RAO) is a vision-threatening disease, primarily affecting patients older than 60 years. Insufficient blood flow in the retinal arteries leading to infarction of the affected retinal tissue is caused by several different mechanisms including embolization, hemorrhage under an atherosclerotic plaque, and thrombus formation [1,2]. Among others, atherosclerosis has been identified as a major risk factor for RAO [1].

Atherosclerosis is a chronic low-grade inflammatory disease [3]. Both pro- and anti-inflammatory cytokines have been shown to modulate atherogenesis [4,5]. Interleukin 10 (IL-10), a pleiotropic, anti-inflammatory cytokine, is synthesized by several cell types including macrophages and T lymphocytes [6]. It exerts antiatherogenic effects by suppressing the production of both pro-inflammatory cytokines and matrix metalloproteinases, and by downregulating the expression of tissue factor (TF) and adhesion molecules [6-12]. Further evidence for the role of

IL-10 in atherogenesis comes from animal experiments, demonstrating that increased IL-10 expression leads to reduced formation of early and advanced atherosclerotic lesions, whereas IL-10 deficiency is associated with enhanced atherogenesis [10,13-19].

In humans, IL-10 production is to a large extent genetically determined [20,21]. The IL10 gene is located at chromosome 1q31-q32. A [TCATA] haplotype formed by polymorphisms at position -3575, -2763, -1082, -819 and -592 in the promoter region of the IL10 gene has been associated with increased IL-10 expression [22]. Due to linkage disequilibrium, the presence of this haplotype can be fully determined by the analysis of the IL10 -592C>A gene polymorphism (rs1800872). The [TCATA] haplotype is identified by the presence of the IL10 -592A-allele, while the -592C-allele indicates its absence [22,23].

The purpose of the present study was to investigate a hypothesized association between the haplotype-tagging IL10 -592C>A polymorphism and the presence of RAO.

## Methods

The present retrospective case-control study consisted of 194 patients with RAO and 257 control subjects. All participants, who were seen at the local department of ophthalmology between October 1998 and July 2006, gave written informed consent prior to being enrolled. The study was conducted in accordance with the guidelines of the National Gene Technology Act and the local Ethics Committee.

All participants underwent an ophthalmologic examination that included visual acuity tests as well as, slit-lamp and fundus examinations. The diagnosis of RAO was made by fundus examination in which superficial retinal whitening was found in the distribution of the affected retinal artery. Since second eye involvement occurred in two patients, 93 eyes were diagnosed with central retinal artery occlusion (CRAO), while branch retinal artery occlusion (BRAO) was present in 103 eyes. Patients with RAO due to giant cell arteritis or other types of vasculitis were considered ineligible for participation.

The control group was comprised of patients who had been seen at the local department of ophthalmology for reasons other than RAO. Those with a history of retinal vascular occlusion or vasculitis were deemed ineligible.

Arterial hypertension was defined by systolic blood pressure greater or equal to 140 mm Hg and/or diastolic blood pressure greater or equal to 90 mm Hg, and/or the current intake of antihypertensive drugs. Subjects were classified as diabetics, if they were being treated for insulin- or non-insulin-dependent diabetes. Hypercholesterolemia was defined by the intake of lipid-lowering drugs and/or a fasting plasma cholesterol level >200 mg/dL. Patients were further classified as current or non-smokers.

### Genotype determination

Genomic DNA was isolated from peripheral blood lymphocytes by standard methods and stored at -20 °C. Genotypes of the IL10 -592C>A polymorphism were determined by a 5'-exonuclease assay (TaqMan) using a procedure described by Langsenlehner, et al. [24]. Primer and probe sets were designed and manufactured using Applied Biosystems "Assay-by-Design" custom service (Applera, Vienna, Austria). The polymerase chain reaction (PCR) was performed in a Primus 96 plus thermal cycler (MWG Biotech AG, Germany) using a total volume of 5 μl containing 2.5 μl SuperHot-Master-Mix (Bioron GmbH, Ludwigshafen, Germany), 0.125 μl 40x Assay-by-Design mix (Applera), 0.375 μl H_2_O and 2 μl DNA. Reactions were overlaid with 15 μl mineral oil. Cycling parameters were as follows: 1 min 94 °C for primary denaturation, followed by 40 cycles of 15 s 92 °C and 1 min 60 °C. Fluorescence was measured in a lambda Fluoro 320 Plus plate reader (MWG Biotech AG) using excitation/emission filters of 485/530 nm for FAM-labeled probes (IL10 -592A-allele) and 530/572 nm for VIC-labeled probes (IL10 -592C-allele). The data were exported into Excel format and depicted and analyzed as a scatter plot.

### Statistics

SPSS for Windows (release 11.0.1; SPSS, Inc) was used for statistical analyses. Continuous variables were analyzed by Student's t-test and are presented as means±SD. Categorial variables are presented as percentages and were compared by chi-square test. Odds ratios and 95% confidence intervals (CI) were determined by logistic regression analysis. The criterion for statistical significance was p less than or equal to 0.05.

## Results

Clinical characteristics of patients and control subjects are shown in Table 1. Prevalences of arterial hypertension and current-smoking status were significantly higher in patients than among controls.

**Table 1:** Clinical characteristics of retinal artery occlusion patients and controls.

	Patients with RAO (n=194)	Controls (n=257)
Males	122 (62.9)	145 (56.4)
Females	72 (37.1)	112 (43.6)
Mean age (years±SD)	68.7±11.8	69.1±10.9
Arterial hypertension	154 (79.4)	155 (60.3)*
Diabetes mellitus	27 (13.9)	22 (8.6)
Hypercholesterolemia	139 (71.6)	162 (63.0)
Current smoker	47 (24.2)	28(10.9)*

Clinical characteristics of retinal artery occlusion (RAO) patients and controls. Prevalences of arterial hypertension and current smoking status were significantly higher in patients than among control subjects. Numbers are given as n (%); asterisk (*) indicates p<0.001.

Table 2 presents the genotype distribution of the IL10 -592C>A polymorphism in patients and control subjects. Carriers of the IL10 -592A-allele (AA+CA genotypes) were found significantly more often in the control group than among patients with RAO (p=0.008). Carriage of the IL10 -592A-allele yielded an odds ratio of 0.60 (95% CI: 0.41-0.87; p=0.008) for RAO, and remained significant after adjusting for age, sex, arterial hypertension, hypercholesterolemia, smoking status and diabetes mellitus (odds ratio: 0.65; 95% CI: 0.44-0.97; p=0.036).

**Table 2:** Distribution of IL10 -592C>A genotypes in retinal artery occlusion patients and controls.

	Patients with RAO (n=194)	Controls (n=257)	p
CC	124 (63.9)	132 (51.4)	
CA	63 (32.5)	107 (41.6)	
AA	7 (3.6)	18 (7.0)	0.008 *

Distribution of IL10 -592C>A genotypes in retinal artery (RAO) patients and controls. Carriers of the IL10 -592A-allele (AA+CA genotypes) were found significantly more often in the control group than among patients with retinal artery occlusion. Numbers for genotypes are given as n (%). Data were compared by chi square test asterisk (*; AA+CA) versus CC.

In a subgroup analysis, prevalences of the CC, CA and AA genotypes did not significantly differ between eyes with CRAO (61.3%, 36.6%, and 2.2%, respectively) and those with BRAO (67.0%, 28.2%, and 4.9%, respectively).

No deviation from the genotype distribution predicted by the Hardy-Weinberg equilibrium was observed in either patients or controls, and allelic frequencies in the control group were similar to those previously reported by other investigators [24,25].

## Discussion

The production of IL-10 has been shown to be under tight genetic control with heritability estimates up to 74% [20]. The IL10 [TCATA] haplotype formed by five polymorphisms in the promoter region of the IL10 gene has been associated with increased IL-10 synthesis [22]. Due to linkage disequilibrium, the absence or presence of the IL10 [TCATA] haplotype can be unequivocally determined by analysis of the IL10 -592C>A polymorphism [22,23].

To the best of our knowledge, the present study is the first to investigate the haplotype-tagging IL10 -592C>A polymorphism in patients with RAO. Genotyping was performed in 194 patients and 257 control subjects. Carriers of the -592A-allele (AA+CA), which indicates the presence of the high IL-10-producing [TCATA] haplotype, were found significantly more often in controls than among patients with RAO. A subgroup analysis revealed a similar genotype distribution between eyes with BRAO and CRAO, indicating that this polymorphism is a risk factor for both types of RAO. In a logistic regression analysis, after adjusting for age, sex, arterial hypertension, hypercholesterolemia, diabetes mellitus, and smoking habits the presence of the IL10 -592A-allele was associated with an odds ratio of 0.65 for RAO, suggesting a protective effect against RAO.

Besides arterial hypertension and cigarette smoking, which are known to initiate or promote atherogenesis [3], atherosclerosis itself plays a major role in the pathogenesis of RAO [1,2]. Since increased IL-10 expression has been shown to reduce the formation of atherosclerotic lesions [10,13-19], our finding that the IL10 [TCATA] haplotype exerts a protective effect against RAO seems biologically plausible. Among other mechanisms, IL-10 mediates antiatherogenic pathways by suppressing the production of pro-inflammatory cytokines such as interleukin 1 beta (IL-1β), interleukin 12 (IL-12), tumor necrosis factor alpha (TNF-α) and interleukin-6 (IL-6) [6-8]. Interestingly, an IL-6 promoter polymorphism has recently been reported as a novel risk factor for RAO [26]. This further suggests that genetic influences on both pro- and anti-inflammatory cytokines contribute to the risk of RAO.

As a potential limitation of our study, IL-10 plasma concentrations were not determined. The purpose of our study, however, was to investigate the role of a haplotype-tagging genetic polymorphism, since in contrast to plasma cytokine concentrations, genetic variants do not change over life-time. Beside the [TCATA] haplotype, other polymorphisms in the IL10 gene might also influence IL-10 expression and thereby affect RAO risk. Further studies focusing on these gene variants may provide additional insight in the role of IL10 in RAO.

In conclusion, our study demonstrates that the IL10 [TCATA] haplotype, indicated by the presence of the IL10 -592A-allele, may be associated with a protective effect against RAO. Yet, large prospective studies are warranted to confirm the contribution of these IL10 promoter gene polymorphisms to the risk of RAO.
